# Retropharyngeal lymph node metastasis on N stage of nasopharyngeal carcinoma

**DOI:** 10.1371/journal.pone.0253424

**Published:** 2021-06-17

**Authors:** Xin-Bin Pan, Shi-Ting Huang, Song Qu, Kai-Hua Chen, Yan-Ming Jiang, Xiao-Dong Zhu

**Affiliations:** Department of Radiation Oncology, Guangxi Medical University Cancer Hospital, Nanning, Guangxi, P.R. China; Yale-New Haven Hospital, UNITED STATES

## Abstract

**Purposes:**

To evaluate retropharyngeal lymph node metastasis on N stage of nasopharyngeal carcinoma (NPC).

**Methods:**

NPC patients were extracted from the Surveillance, Epidemiology, and End Results database between 2004 and 2016. Pathologically confirmed patients with complete data of retropharyngeal lymph node metastasis were investigated. The included patients were divided into N1a and N1b groups. Overall survival (OS) and cancer-specific survival (CSS) were assessed using the Kaplan–Meier method and propensity score matching (PSM) analyses.

**Results:**

This retrospective cohort study examined 759 patients: 70 who were stage N1a and 689 who were stage N1b. Before PSM, N1a group was associated with similar 5-year OS (77.7% vs. 72.4%; *P* = 0.15) and CSS (85.6% vs. 79.9%; *P* = 0.09) compared to N1b group. After PSM, a similar OS (75.0% vs. 60.7%; *P* = 0.12) was found between the radiotherapy and chemoradiotherapy groups. However, N1a group showed a better 5-year CSS (83.8% vs. 71.1%; *P* = 0.04) compared to N1b group. Stage N1b was an independent risk prognostic factor for CSS (hazard ratio = 2.54, 95% confidence interval: 1.02–6.34; *P* = 0.04).

**Conclusions:**

OS was not different between N1a and N1b groups. Retropharyngeal lymph node metastasis defined as stage N1 of the 8^th^ edition American Joint Committee on Cancer staging system is reasonable.

## Introduction

An accurate staging system is crucial for clinicians to formulate treatment plans and evaluate treatment outcomes. According to the 7^th^ edition American Joint Committee on Cancer (AJCC) staging system, unilateral and bilateral retropharyngeal nodes metastasis was coded to stage N1 for NPC [1]. In contrast, retropharyngeal nodes metastasis was classified as stage N1a in the 2008 Chinese edition. Unilateral cervical lymph nodes ≤ 3 cm above caudal border of cricoid cartilage was classified as stage N1b. It was reported that the Chinese 2008 N1 classification was superior in predicting the 5-year distant metastasis-free survival compared to the stage N1 of the AJCC 7^th^ edition [2].

However, Pan et al. [3] found that the 5-year distant metastasis-free survival were not different between stage N1a and N1b of the 2008 Chinese edition. With the purpose of simplification of unnecessary subgroups, the 8^th^ edition AJCC staging system defied stage N1 as a whole group including retropharyngeal nodes metastasis and unilateral cervical lymph nodes ≤ 6 cm above caudal border of cricoid cartilage. This raises a question of whether overall survival (OS) between stage N1a and N1b is different. Thus, we conducted this retrospective cohort study to assess the difference of OS between stage N1a and N1b.

## Materials and methods

### Patients

This study investigated NPC patients in the Surveillance, Epidemiology, and End Results (SEER) database from 2004 to 2016. Patient selection was based on the following protocol. (1) Pathologically confirmed NPC were investigated. (2) The International Classification of Diseases for Oncology third edition was used to identified histology. World Health Organization (WHO) type I codes 8070 and 8071 (keratinizing squamous cell carcinoma), type II codes 8072 and 8073 (differentiated nonkeratinizing carcinoma); and type III codes 8020, 8021, and 8082 (undifferentiated nonkeratinizing carcinoma) were used to identify the patients. (3) Patients with incomplete clinical information were excluded. (4) Patients with stage T1-4N1M0 were included. (5) Patients receiving radiotherapy were included. (6) Patients without definite data of retropharyngeal nodes metastasis were excluded. Included patients were divided into two groups based on the status of retropharyngeal nodes metastasis: N1a and N1b according to the 2008 Chinese edition [2].

### Treatment endpoints

OS was the primary endpoint. OS was defined as the time from diagnosis to death as a result of any cause. Cancer-specific survival (CSS) was the secondary endpoint. CSS was defined as the time from diagnosis to death attributed to NPC.

### Statistical analysis

Continuous characteristic of age was compared using Student’s t-test. Categorical variables of race, sex, WHO classification, tumor grade, T stage, and chemotherapy were analyzed by using the χ2 test or Fisher exact test.

A matched case-control analysis was conducted using propensity score matching (PSM) to reduce the influence of selection bias on the comparison of efficacy between N1a and N1b groups. A logistic regression model was established in which N1a group was considered the dependent variable in the process of calculating the propensity scores. One-to-one matching without replacement was completed using the nearest-neighbor match on the logit of the propensity score for confounding factors with a caliper of 0.01.

Cumulative survival curves of 5-year OS and CSS were calculated using the Kaplan-Meier method. Differences between survival curves were compared using the log-rank test. The hazard ratios (HRs) and 95% confidence intervals (CIs) for OS and CSS were estimated with the use of a stratified Cox regression model, with the stratification factors of age, race, sex, WHO classification, tumor grade, T stage, and chemotherapy.

All statistical analyses were performed using SPSS Statistics Version 26.0 software (IBM Co., Armonk, NY, USA) and R software version 4.0.3 (http://www.R-project.org). P values were two-tailed. Values of P < 0.05 were considered statistically significant.

## Results

### Patient characteristics

**Fig 1:**
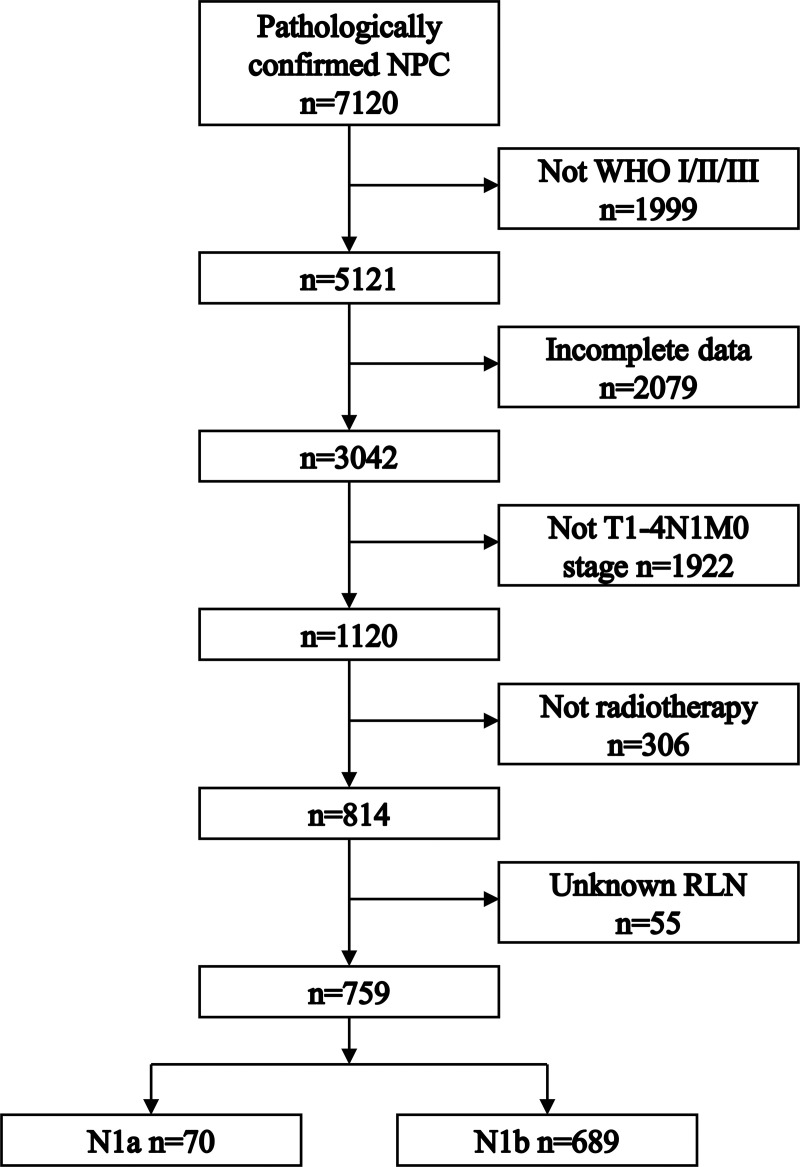
Flowchart depicting patient selection.

Fig 1 shows the process of patient selection. This study included 759 NPC patients. Table 1 shows the patient characteristics. Baseline characteristics were different in the variables of age and T stage before PSM. After PSM, 62 patients in the N1a group and 62 patients in the N1b group were matched. Patient characteristics were well balanced across all covariates after PSM. The median follow-up times were 50 months and 55 months for the N1a group and N1b group.

**Table 1:** Patient characteristics.

	The unmatched cohort		*P*	The PSM cohort		*P*
	N1a (n = 70)	N1b (n = 689)		N1a (n = 62)	N1b (n = 62)	
Age (median)	51	55	0.03	51	53	0.51
IQR	43–59	46–63		43–60	43–62	
Sex			0.28			0.83
male	54 (77.1%)	484 (70.2%)		46 (74.2%)	48 (77.4%)	
female	16 (22.9%)	205 (29.8%)		16 (25.8%)	14 (22.6%)	
Race:			0.19			0.82
Asian	35 (50.0%)	269 (39.0%)		28 (45.2%)	28 (45.2%)	
Black	7 (10.0%)	73 (10.6%)		7 (11.3%)	5 (8.1%)	
White	28 (40.0%)	347 (50.4%)		27 (43.5%)	29 (46.8%)	
Grade:			0.10			0.89
I	0 (0.0%)	15 (2.2%)		0 (0.0%)	1 (1.6%)	
II	5 (7.2%)	98 (14.2%)		5 (8.1%)	4 (6.5%)	
III	26 (37.1%)	283 (41.1%)		26 (41.9%)	24 (38.7%)	
IV	39 (55.7%)	293 (42.5%)		31 (50.0%)	33 (53.2%)	
Pathology:			0.06			0.83
WHO I	18 (25.7%)	277 (40.2%)		18 (29.0%)	15 (24.2%)	
WHO II	25 (35.7%)	205 (29.8%)		23 (37.1%)	25 (40.3%)	
WHO III	27 (38.6%)	207 (30.0%)		21 (33.9%)	22 (35.5%)	
T stage:			0.03			0.44
T1	13 (18.6%)	248 (36.0%)		13 (21.0%)	15 (24.2%)	
T2	23 (32.9%)	174 (25.3%)		20 (32.3%)	18 (29.0%)	
T3	20 (28.6%)	140 (20.3%)		18 (29.0%)	12(19.4%)	
T4	14 (20.0%)	127 (18.4%)		11 (17.7%)	17 (27.4%)	
Chemotherapy:			0.78			0.99
no	4 (5.7%)	36 (5.2%)		4 (6.45%)	3 (4.84%)	
yes	66 (94.3%)	653 (94.8%)		58 (93.5%)	59 (95.2%)	

IQR: interquartile range. WHO: World Health Organization. PSM: propensity score matching.

### Survival analysis before propensity score matching

**Fig 2:**
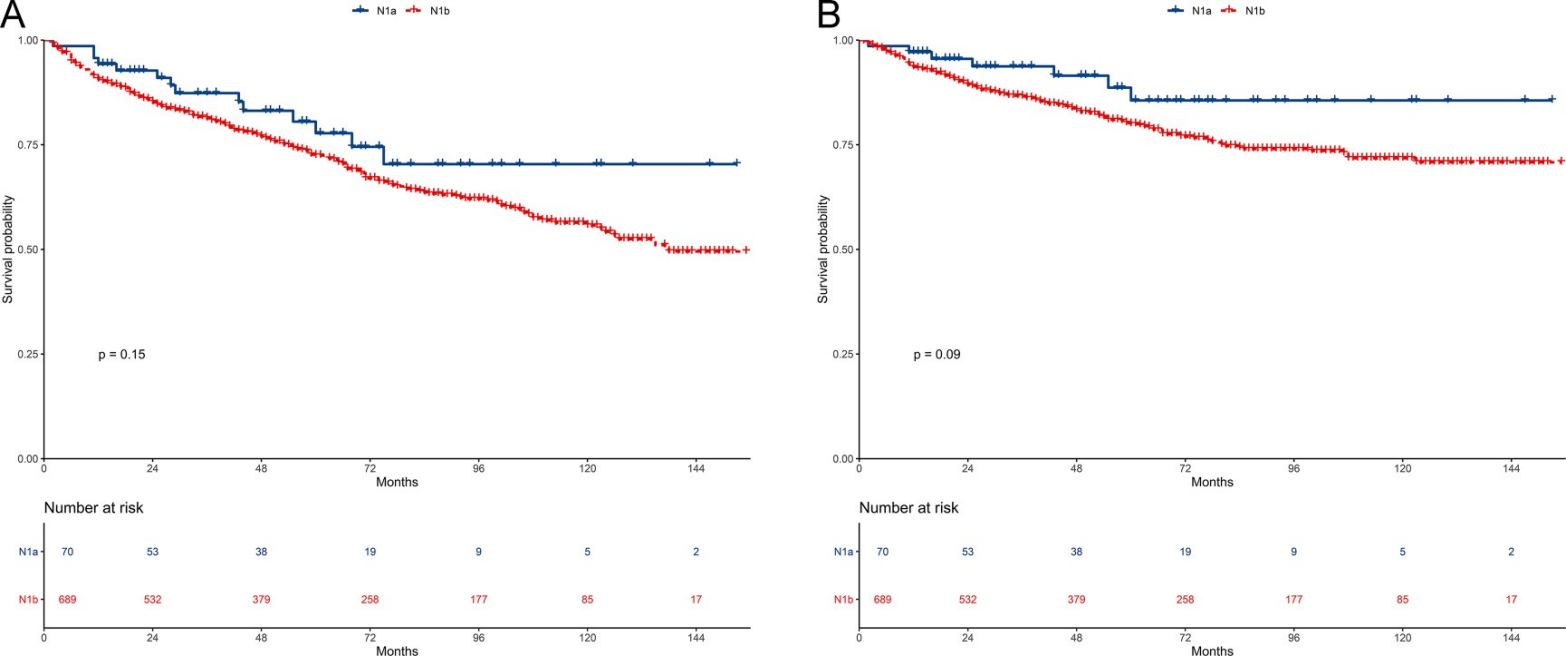
Survival between N1a and N1b groups in the unmatched cohort. (A) Overall survival. (B) Cancer-specific survival.

**Fig 3:**
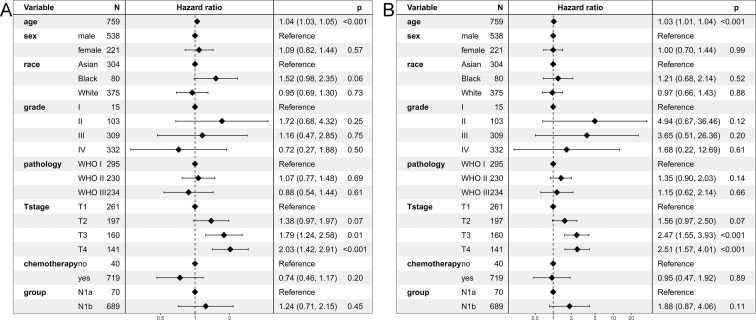
Multivariate regression analysis of prognostic factors for survivals in the unmatched cohort. (A) Overall survival. (B) Cancer-specific survival.

The 5-year OS of the N1a and N1b groups was 77.7% and 72.4% (*P* = 0.15). OS was comparable between the two groups (Fig 2A). The stratified HR of N1b group was 1.24 (95% CI: 0.71–2.15; *P* = 0.45) in the multivariate regression analysis (Fig 3A).

The 5-year CSS of the N1a and N1b groups was 85.6% and 79.9% (*P* = 0.09). CSS of the N1a group was similar than that of N1b group (Fig 2B). The stratified HR of N1b group was 1.88 (95% CI: 0.87–4.06; *P* = 0.11) in the multivariate regression analysis (Fig 3B).

### Survival analysis after propensity score matching

**Fig 4:**
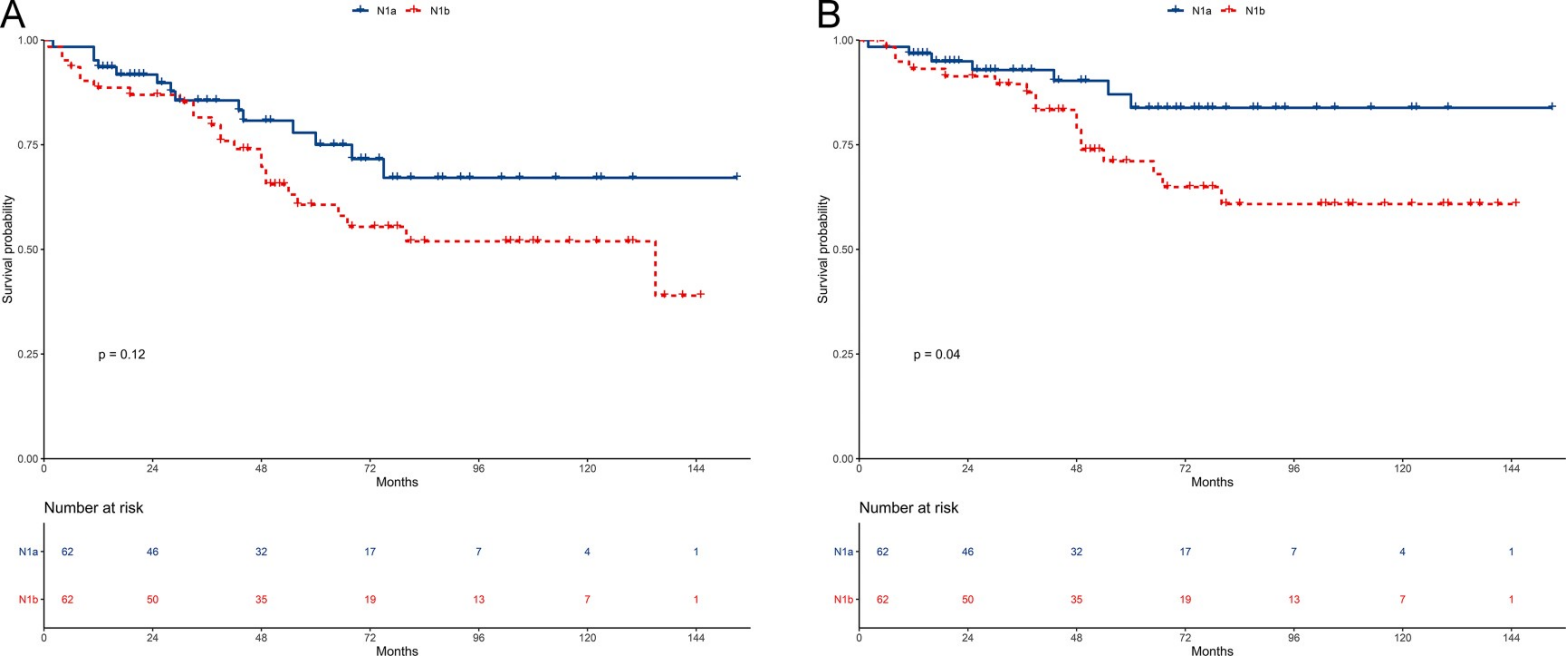
Survival between N1a and N1b groups in the propensity-matched cohort. (A) Overall survival. (B) Cancer-specific survival.

**Fig 5:**
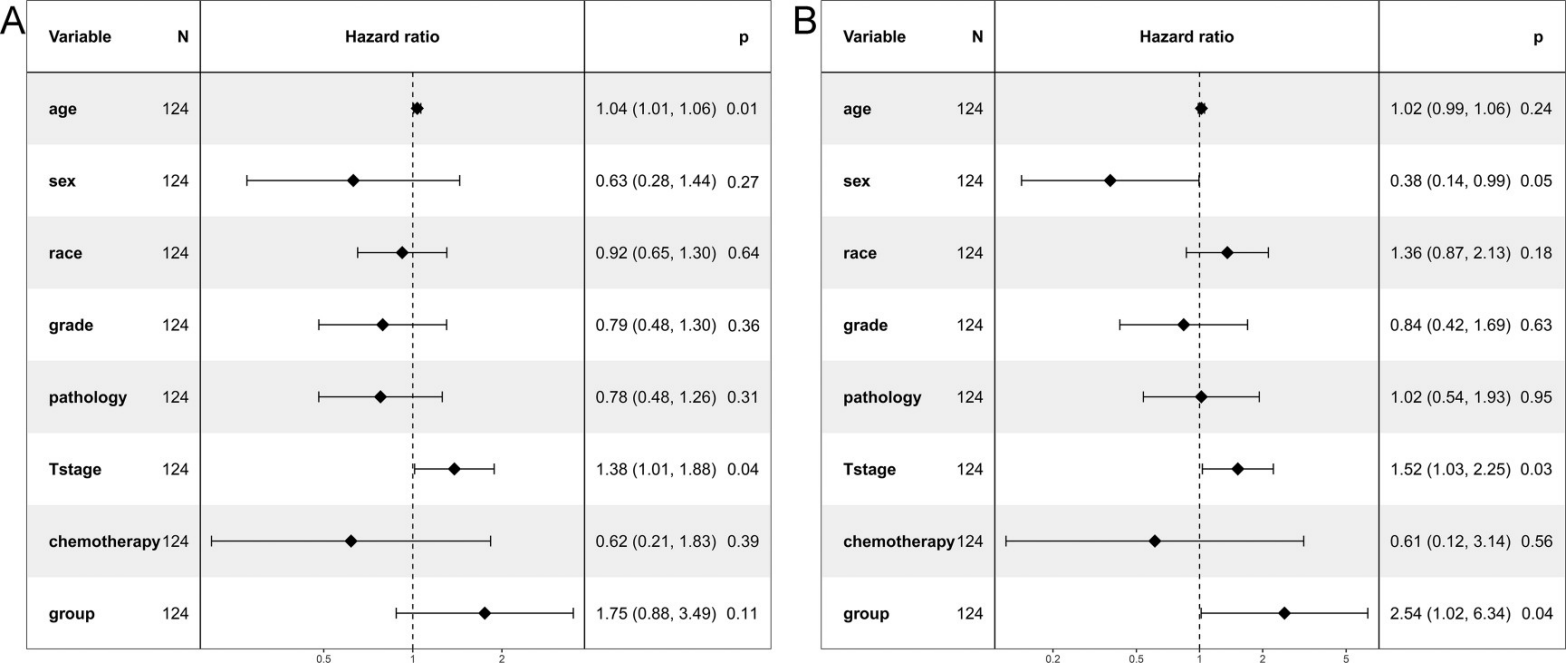
Multivariate regression analysis of prognostic factors for survivals in the propensity-matched cohort. (A) Overall survival. (B) Cancer-specific survival.

In the matched cohort, the 5-year OS of the N1a and N1b groups was 75.0% and 60.7% (*P* = 0.12). OS was comparable between the two groups (Fig 4A). The stratified HR of N1b group was 1.75 (95% CI: 0.88–3.49; *P* = 0.11) in the multivariate regression analysis (Fig 5A).

The 5-year CSS of the N1a and N1b groups was 83.8% and 71.1% (*P* = 0.04). CSS of the N1b group was worse than that of N1a group (Fig 4B). The stratified HR of N1b group was 2.54 (95% CI: 1.02–6.34; *P* = 0.04) in the multivariate regression analysis (Fig 5B). N1b group was an independent risk prognostic factor of CSS.

## Discussion

Our study evaluated the prognostic value of retropharyngeal nodes metastasis for NPC. The results showed that no difference for OS was found between stage N1a and N1b groups. Stage N1b was not an independent prognostic factor for OS. These results suggested that retropharyngeal nodes metastasis, regardless of its laterality, classified as N1 disease is reasonable for NPC of the 8^th^ edition.

It is reported that neck lymph node metastasis spreads in an orderly manner down the neck in NPC [4, 5]. As the first echelon lymph node of NPC [6], retropharyngeal nodes metastasis is very common. The incidence of retropharyngeal nodes metastasis of NPC is approximately 70% [7, 8]. Thus, it is crucial to assess the prognosis and appropriate classification of staging system for retropharyngeal nodes metastasis.

Retropharyngeal nodes metastasis was firstly incorporated into the 7^th^ edition AJCC staging system for NPC [1]. Previous retrospective studies reported that retropharyngeal nodes metastasis alone had a similar distant-metastasis free survival compared with stage N1 [9, 10]. Thus, retropharyngeal nodes metastasis was classified as stage N1 in the 7^th^ edition. However, the classification of the 7^th^ edition was mainly based on two-dimensional conventional radiotherapy (2D-CRT). It is reported that intensity-modulated radiation therapy (IMRT) improves survivals in NPC patients compared to two-dimensional conventional radiotherapy (2D-CRT) [11]. Whether it is still reasonable for retropharyngeal nodes metastasis to be classified as stage N1 in the IMRT era is still unclear.

In IMRT era, it was reported that significant differences of distant metastasis-free survival were observed between stage N1, N0, and N2 according to the 7^th^ edition [12, 13]. Moreover, Pan et al. [3] revealed that distant metastasis-free survivals between N1a and N1b were not different. Similarly, a previous study also revealed that no differences were observed in the 5-year disease free survival (*P* = 0.091) and distant metastasis-free survival (*P* = 0.058) between N1a and N1b groups in the IMRT era [14]. Thus, retropharyngeal nodes metastasis was still defied as stage N1 in the 8^th^ edition AJCC staging system with the purpose of simplification of unnecessary subgroups by elimination. The current study further indicated that OS was similar between N1a and N1b groups both before and after PSM. These results suggested the reasonability of the stage N1 of the 8^th^ edition.

However, our study found that the differences between N1a and N1b groups in CSS were statistically significant after PSM. This result of our study was not consistent with other studies [10, 14]. A possible explanation was that the sample size of N1a group was small in our study. The small sample size might significantly reduce the statistical power of the analysis. Moreover, data of distant failure could not be extracted due to the limitations of SEER database. This study could not assess the distant-metastasis free survival between N1a and N1b groups. Therefore, it was unknown whether the unfavorable CSS in the N1b group was due to distant failure or not. There is a need to investigate the feasibility of classifying retropharyngeal nodes metastasis as N1a disease in future by a larger cohort study.

In the 2008 Chinese edition, stage N1a was defined as retropharyngeal nodes metastasis. Stage N1b was defined as unilateral cervical lymph nodes ≤ 3 cm above caudal border of cricoid cartilage. However, stage N1a and N1b were only incorporated into the stage II group (T1N1a-1bM0, T2N0-1bM0) of the 2008 Chinese edition [2]. Previous study showed that distant metastasis-free survival and OS were not different among subgroups of stage II patients [15]. The classification of stage N1 of the 2008 Chinese edition had no impact on prognosis. Thus, it is reasonable that N1a and N1b were classified as a whole group in the 8^th^ edition.

This study had some limitations. First, a total of 70 (9.22%) patients were included in the N1a group. The small sample size of the N1a group might have been insufficient for statistical analysis compared to that of the N1b group. This may have significantly reduced the statistical power of the analysis. Second, data regarding the radiotherapy technique were not extracted in this study given the limitations of the SEER database. Although intensity-modulated radiation therapy was reported to be superior to two-dimensional conventional radiotherapy [11, 16], several studies have revealed that no difference was observed in OS between IMRT and 2DCRT [17, 18]. Thus, the different radiotherapy techniques used in this study might not have had an impact on the results. Third, we could not assess the impact of chemotherapy regimen on survivals due to the lacking of chemotherapy information. However, we performed several analytic techniques, including multivariable adjustment and PSM to control potential biases. The multivariable analysis before and after PSM both revealed that chemotherapy was not an independent prognostic factor for OS and CSS.

## Conclusions

In conclusion, the current retrospective study showed that OS was not different between N1a and N1b groups. Retropharyngeal lymph node metastasis defined as stage N1 is reasonable.

## Supporting information

S1 Checklist STROBE statement—Checklist of items that should be included in reports of observational studies. (DOCX)

S1 Dataset (XLSX)
